# PREVENT Equation Performance in Asian and Native Hawaiian and Other Pacific Islander Groups

**DOI:** 10.1001/jamanetworkopen.2025.56915

**Published:** 2026-02-12

**Authors:** Michael Au, Yiyi Zhang, Matt M. Zhou, Soon Kyu Choi, Hui Zhou, Teresa N. Harrison, Matthew T. Mefford, Ming-Sum Lee, Eugene Yang, Nilay S. Shah, Kristi Reynolds, Jaejin An

**Affiliations:** 1 Kaiser Permanente Bernard J. Tyson School of Medicine, Pasadena, California; 2 Division of General Medicine, Columbia University Irving Medical Center, New York, New York; 3 Department of Research and Evaluation, Kaiser Permanente Southern California, Pasadena, California; 4 Department of Health Systems Science, Kaiser Permanente Bernard J. Tyson School of Medicine, Pasadena, California; 5 Department of Cardiology, Kaiser Permanente Los Angeles Medical Center, Los Angeles, California; 6 Division of Cardiology, University of Washington School of Medicine, Seattle; 7 Department of Medicine, Northwestern University Feinberg School of Medicine, Chicago, Illinois

## Abstract

**Importance:**

In 2023, the PREVENT (Predicting Risk of CVD Events) equations were introduced to estimate 10-year risk of total cardiovascular disease (CVD). However, their accuracy in individual Asian or Native Hawaiian and Other Pacific Islander ethnic groups remains unknown.

**Objective:**

To evaluate the risk prediction accuracy of the PREVENT base and full equations in Asian and Native Hawaiian and Other Pacific Islander ethnic groups.

**Design, Setting, and Participants:**

This retrospective cohort study was conducted among adults aged 30 to 79 years without CVD who self-reported as being non-Hispanic White, Asian, or Native Hawaiian and Other Pacific Islander and were active Kaiser Permanente Southern California members as of September 30, 2009. Participants were followed up through 2019. Non-Hispanic Asian adults were further disaggregated into ethnic groups. Analysis was performed between February and June 2025.

**Main Outcomes and Measures:**

The main measures were the PREVENT base equation (age, total and high-density lipoprotein cholesterol, systolic blood pressure, body mass index, estimated glomerular filtration rate, diabetes, smoking, and lipid and antihypertensive medication) and the PREVENT full equation (base plus hemoglobin A_1c_, urine albumin-creatinine ratio, and Social Deprivation Index). The main outcome was the 10-year incidence of total CVD, atherosclerotic CVD, and heart failure. Estimated risks were compared with observed events using the Harrell C index and mean calibration (predicted to observed event ratios).

**Results:**

The study cohort consisted of 542 848 adults, including 424 277 non-Hispanic White adults (mean [SD] age, 55.6 [11.8] years; 235 722 [55.6%] female), 110 855 non-Hispanic Asian adults (mean [SD] age, 52.5 [11.9] years; 66 292 [59.8%] female), and 7716 non-Hispanic Native Hawaiian and Other Pacific Islander adults (mean [SD] age, 51.4 [11.9] years; 4 398 [57.0%] female). A total of 31 556 CVD events occurred during 10 years. For total CVD, the PREVENT base equation demonstrated good discrimination across non-Hispanic White (C index, 0.764; 95% CI, 0.761-0.767), non-Hispanic Asian (C index, 0.773; 95% CI, 0.765-0.779) and non-Hispanic Native Hawaiian and Other Pacific Islander (C index, 0.757; 95% CI, 0.733-0.780) groups. Among Asian ethnic groups, C indexes for the PREVENT base equation ranged from 0.738 (95% CI, 0.701-0.774) in Vietnamese adults to 0.806 (95% CI, 0.787-0.826) in Chinese adults. The PREVENT full equations showed consistent results. The PREVENT base and full equations generally overestimated total CVD, atherosclerotic CVD, and heart failure risk in non-Hispanic Asian (mean calibration, 0.96-1.33) and underestimated risk in non-Hispanic Native Hawaiian and Other Pacific Islander (mean calibration, 0.74-0.96) and non-Hispanic White (mean calibration, 0.63-1.03) populations.

**Conclusions and Relevance:**

In this retrospective cohort study, the PREVENT base and full equations demonstrated overall strong performance in predicting 10-year cardiovascular risk. However, notable differences were observed across disaggregated Asian and Native Hawaiian and Other Pacific Islander ethnic groups, underscoring the importance of recognizing heterogeneity within these populations when applying risk prediction models.

## Key Points

**Question:**

How well do the PREVENT base and full equations predict 10-year risk of cardiovascular disease (CVD) in non-Hispanic Asian and Native Hawaiian and Other Pacific Islander ethnic groups?

**Findings:**

In this cohort study of 542 848 adults, the PREVENT equations demonstrated good overall discrimination and calibration for total CVD, atherosclerotic CVD, and heart failure in non-Hispanic Asian and Native Hawaiian and Other Pacific Islander groups. However, performance varied among disaggregated Asian ethnic groups.

**Meaning:**

Despite overall strong performance, the observed heterogeneity within Asian and Native Hawaiian and Other Pacific Islander ethnic groups highlights the need for tailored CVD risk prediction models that account for racial and ethnic disparities.

## Teaser

This cohort study of non-Hispanic White, Asian, or Native Hawaiian and Other Pacific Islander adults evaluates the cardiovascular disease and heart failure risk prediction accuracy of the PREVENT base and full equations.

## Introduction

Cardiovascular disease (CVD) remains the leading cause of morbidity and mortality in the US^1^; as such, multisociety CVD prevention guidelines recommend the use of CVD risk estimation models to help guide the intensity of preventive efforts.^2,3,4^ In 2023, the American Heart Association developed the Predicting Risk of CVD Events (PREVENT) equations to improve estimation of CVD risk. Unlike the previous pooled cohort equations, the PREVENT equations do not incorporate race as a predictor. Instead, the PREVENT base equation accounts for traditional risk factors, such as total cholesterol (TC), high-density lipoprotein cholesterol (HDL-C), systolic blood pressure (SBP), diabetes, smoking, and antihypertensive use, as well as body mass index (BMI [calculated as weight in kilograms divided by height in meters squared], for heart failure [HF] prediction only), estimated glomerular filtration rate (eGFR), and lipid-lowering therapy. The PREVENT full equation expands on the PREVENT base equation by optionally accounting for hemoglobin A_1c_, urine albumin-creatinine ratio (UACR), and Social Deprivation Index (SDI) based on zip code. The novel inclusion of UACR and hemoglobin A_1c_ reflect emerging evidence of their strong associations with cardiovascular outcomes^5,6^; similarly, the SDI was used as a widely available surrogate for social determinants of health, which are an important predictor in the development of CVD.^7^ In addition, the PREVENT equations provide risk prediction for total CVD and its individual components of atherosclerotic cardiovascular disease (ASCVD) and HF.^8^

Although the PREVENT equations were developed by incorporating contemporary data from diverse cohorts, Asian and Native Hawaiian and Other Pacific Islander participants represented less than 3% of the original derivation sample, despite accounting for approximately 6% of the US population.^9^ Only 1 study recently evaluated the accuracy of the PREVENT equations in disaggregated Asian populations in the US^10^; therefore, further validation is needed. Moreover, Asian ethnic groups are often combined together into a single Asian race category and grouped together with Native Hawaiian and Other Pacific Islander populations, despite substantial heterogeneity in cardiovascular risk factors, disease prevalence, and outcomes across individual groups.^11,12,13^ East Asian (such as Chinese), Southeast Asian (such as Filipino), South Asian (such as Indian), and Native Hawaiian and Other Pacific Islander populations have distinct patterns of metabolic syndrome, hypertension, diabetes, and lipid abnormalities, leading to differences in CVD risk.^14,15^ Thus, this study aims to evaluate total CVD, ASCVD, and HF risk prediction accuracy of the PREVENT base and full equations in Asian and Native Hawaiian and Other Pacific Islander ethnic groups within a large, integrated US health care delivery system. Given known differences in cardiovascular profiles and risk patterns across Asian and Native Hawaiian and Other Pacific Islander subgroups, we hypothesized that the PREVENT equations’ performance would vary across these populations.

## Methods

The retrospective cohort study protocol was approved by the Kaiser Permanente Southern California (KPSC) institutional review board and waiver of consent was granted because the research involved no more than minimal risk to research participants and could not practically be carried out without the waived consent. The study followed the Strengthening the Reporting of Observational Studies in Epidemiology (STROBE) reporting guideline.

### Study Setting

The study population was from KPSC, a large US integrated health care system. KPSC currently provides care to 4.9 million members at 16 medical centers and more than 200 medical offices located across Southern California. The membership is diverse and broadly representative of Southern California’s general population. We used KPSC electronic health records, which capture all interactions with the health care system and are available for research.

### Study Cohort

**Figure 1.**
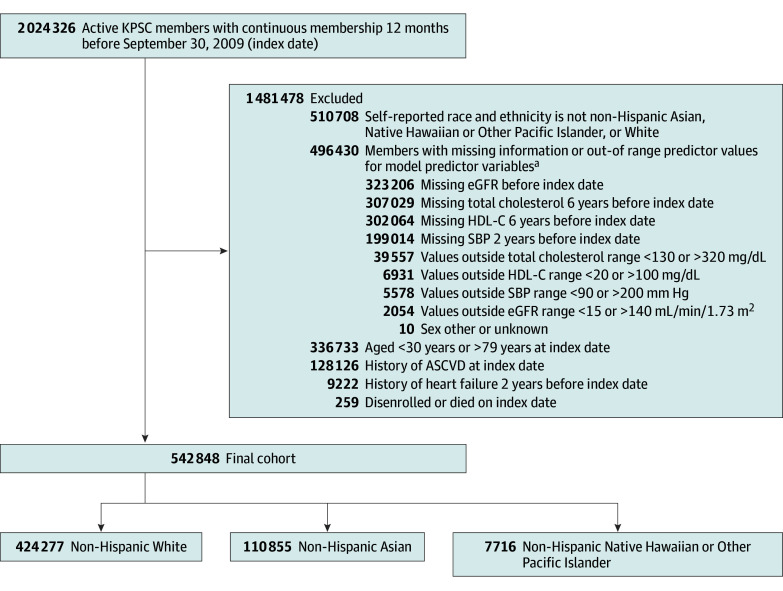
Cohort Flowchart This study included active Kaiser Permanente Southern California (KPSC) members with self-reported race and ethnicity of Non-Hispanic Asian, Native Hawaiian Pacific Islander, or White. ASCVD indicates atherosclerotic cardiovascular disease; eGFR, estimated glomerular filtration rate; HDL-C, high-density lipoprotein cholesterol; SBP, systolic blood pressure. SI conversion factor: To convert cholesterol to millimoles per liter, multiply by 0.0259. ^a^Data are not mutually exclusive.

A retrospective cohort study was conducted among adults aged 30 to 79 years who self-reported as being non-Hispanic Asian, or non-Hispanic Native Hawaiian and Other Pacific Islander, or non-Hispanic White and were actively enrolled as members of KPSC on September 30, 2009 (index date). The identified individuals were followed up for 10 years from the index date until the earliest date of the outcome of interest, disenrollment, death, or end of study (September 30, 2019). Self-reported race and ethnicity data were obtained from the electronic medical record system, and individuals who reported Asian as a race were disaggregated based on self-reported ethnicity into different ethnic groups: Chinese, Filipino, Japanese, Korean, South Asian (eg, Indian, Pakistani, and Bangladeshi), Vietnamese, and Southeast Asian other than Vietnamese (eg, Cambodian, Thai, and Indonesian). The study cohort consisted of individuals without a history of ASCVD or HF before the index date. Individuals missing TC, HDL-C, SBP, or eGFR data at baseline were also excluded to align the study cohort to that of the one assessed by the PREVENT equations (Figure 1). We described demographic and clinical characteristics of excluded individuals to better understand the study population, potential bias, and implications of the findings (eTable 1 in Supplement 1).

### Outcome

The outcomes of interest were incident total CVD, ASCVD, and HF. Total CVD was defined as the composite of ASCVD (myocardial infarction [MI], fatal or nonfatal ischemic stroke, and fatal coronary heart disease [CHD]) and HF. Hospitalizations with a principal discharge diagnosis of MI were identified using *International Classification of Diseases, Ninth Revision (ICD-9) *codes 410.x0 or 410.x1 and *International Statistical Classification of Diseases and Related Health Problems, Tenth Revision (ICD-10) *codes 121.x from hospital discharge records and billing claims. Principal hospital discharge diagnoses indicating ischemic stroke were identified using *ICD-9* codes 433.x1, 434.x1, and 436.xx and *ICD-10* codes I63.x, G46.3, and G46.4. Intracranial hemorrhage was identified by hospital discharge diagnosis with *ICD-9* codes 430, 431, and 432.x and *ICD-10* codes I60.x, I61.x, and I62.x in any positions. Fatal CHD or stroke was identified using *ICD-10* codes I20 to I25 and I60 to I69, respectively, from KPSC sources and National Death Index files. HF was identified by hospital discharge diagnosis with *ICD-9* code 428.x and *ICD-10* code I50.x in any position. Further information regarding the validation of these codes is provided in eTable 2 in Supplement 1.

### Risk Factors

Patient sociodemographic and clinical characteristics, including age, sex, race, ethnicity, and smoking status assessed on index date, were based on a combination of self-report and administrative data. Height and weight data before and closest to the index date were used to calculate BMI. SBP was based on outpatient clinic data within 2 years before the index date, using the value closest to the index date. UACR and hemoglobin A_1c_ were obtained from outpatient laboratory records within 2 years before the index date, whereas HDL-C, TC, and eGFR values were obtained from outpatient laboratory records within 6 years before the index date; for each parameter, the value closest to the index date was used, taking into account the frequency of laboratory measurements. The median (IQR) time between the closest laboratory measurement and the index date was 1 (1-2) year for hemoglobin A_1c_, HDL-C, TC, and eGFR and 1 (1-1) year for UACR. Antihypertensive medications and lipid-lowering therapy were determined from pharmacy dispensing records within 1 year before the index date. Diabetes was defined as having at least 1 primary inpatient discharge diagnosis, 2 or more outpatient diagnoses occurring on separate dates within 12 months before the index date, or 1 or more insulin or an oral hypoglycemic agent dispense along with a diabetes diagnosis. The SDI was derived by mapping members’ addresses to 2008 to 2012 US Census tract data. Deciles of the SDI within the study population were used for analysis. All other risk factors, whether continuous or categorical variables, were used as specified by the PREVENT equation. We excluded individuals with missing or out-of-range values for risk factors necessary for the PREVENT base equation, including TC, HDL-C, SBP, eGFR, and sex. For smoking status on index date, we assumed all missing values (approximately 30%) indicated nonsmoking because most individuals reported being never smokers during follow-up. For variables such as diabetes, antihypertensive medications, and lipid-lowering therapy, we assumed no risk if there were no diagnosis or dispensing records. For variables such as UACR, hemoglobin A_1c_, and SDI, we applied missing indicators as specified by the PREVENT equation.

### Statistical Analysis

Each participant’s 10-year total CVD, ASCVD, and HF risks were estimated by the PREVENT equations. The PREVENT base equation incorporates age, TC, HDL-C, SBP, BMI, eGFR, diabetes status, smoking status, use of antihypertensive medications, and use of lipid-lowering medications. The PREVENT full equation includes all the variables from the PREVENT base equation, in addition to UACR, hemoglobin A_1c_, and zip code to estimate SDI, if available. Model discrimination was assessed using the Harrell C index by estimating the concordance between 10-year PREVENT risk and observed time-to-event data.^16^ Model discrimination differences were assessed by comparing the Harrell C index values from non-Hispanic Asian, non-Hispanic Native Hawaiian and Other Pacific Islander, or disaggregated non-Hispanic Asian ethnic groups with the Harrell C index from the non-Hispanic White group. We selected the non-Hispanic White group as the reference category because of the largest sample size, which provides more stable and reliable estimates. Model calibration was evaluated using mean calibration, which was calculated as the ratio of mean predicted risk to the observed event rates.^17,18^ To further evaluate potential systematic deviations in predicted risk, particularly among individuals at the extreme high- or low-risk groups, calibration curves were constructed by plotting the mean predicted risk vs the observed event rate within deciles of predicted risk.^17,18^ The observed event rate was estimated from the Kaplan-Meier survival analysis. The 95% CIs for these values were estimated using nonparametric bootstrapping for 100 times, with statistical significance defined as a 2-sided *P* < .05. All analyses were performed using SAS Enterprise Guide, version 9.4 (SAS Institute Inc).

## Results

The study cohort consisted of 542 848 adults, including 110 855 non-Hispanic Asian, 7716 non-Hispanic Native Hawaiian and Other Pacific Islander, and 424 277 non-Hispanic White adults. The mean (SD) age was 52.5 (11.9) years for non-Hispanic Asian adults, 51.4 (11.9) years for non-Hispanic Native Hawaiian and Other Pacific Islander adults, and 55.6 (11.8) years for non-Hispanic White adults. There were 66 292 (59.8%) non-Hispanic Asian women and 44 563 (40.2%) non-Hispanic Asian men, 4398 (57.0%) non-Hispanic Native Hawaiian and Other Pacific Islander women and 3318 (43.0%) non-Hispanic Native Hawaiian and Other Pacific Islander men, and 235 722 non-Hispanic White women (55.6%) and 188 555 non-Hispanic White men (44.4%) (Table 1). A total of 24.3% of non-Hispanic Asian adults did not specify their ethnicity (eTable 3 in Supplement 1). Of the non-Hispanic Asian adults, 13 874 (12.5%) were Chinese, 35 490 (32.0%) Filipino, 5226 (4.7%) Japanese, 5843 (5.3%) Korean, 9793 (8.8%) South Asian, 5197 (4.7%) Southeast Asian other than Vietnamese, and 8524 (7.7%) Vietnamese. Japanese adults were the oldest Asian ethnic group, with a mean (SD) age of 59.4 (12.5) years, and had the highest mean total cholesterol level (199.2 mg/dL [to convert to millimoles per liter, multiply by 0.0259]) and prevalence of smoking (249 [4.8%]) and statin use (1970 [37.7%]). In contrast, Filipino adults had the highest mean (SD) BMI (26.4 [4.3]) and prevalence of hypertension (14 537 [41.0%]) and diabetes (6127 [17.3%]). Compared with their non-Hispanic Asian counterparts, non-Hispanic Native Hawaiian and Other Pacific Islander adults had a higher prevalence of smoking (513 [6.6%]), diabetes (1289 [16.7%]), hypertension (2705 [35.1%]), and statin use (2333 [30.2%]).

**Table 1.** Characteristics of Study Population

Characteristic	No. (%) of participants^a^									
	Non-Hispanic White (n = 424277)	Non-Hispanic Asian (n = 110855)	Non-Hispanic Native Hawaiian and Other Pacific Islander (n = 7716)	Disaggregated non-Hispanic Asian ethnic groups (n = 83 947)						
				Chinese (n = 13874)	Filipino (n = 35490)	Japanese (n = 5226)	Korean (n = 5843)	South Asian (n = 9793)	Southeast Asian (n = 5197)	Vietnamese (n = 8524)
Age, mean (SD), y	55.6 (11.8)	52.5 (11.9)	51.4 (11.9)	53.9 (12.1)	52.9 (11.5)	59.4 (12.5)	54.7 (12.7)	50.6 (11.7)	52.6 (11.3)	50.4 (11.4)
Sex^b^										
Female	235 722 (55.6)	66 292 (59.8)	4398 (57.0)	8289 (59.7)	22 118 (62.3)	3240 (62.0)	3568 (61.1)	5134 (52.4)	3115 (59.9)	4836 (56.7)
Male	188 555 (44.4)	44563 (40.2)	3318 (43.0)	5585 (40.3)	13 372 (37.7)	1986 (38.0)	2275 (38.9)	4659 (47.6)	2082 (40.1)	3688 (43.3)
BMI, mean (SD)	29.1 (6.3)	25.5 (4.3)	28.6 (6.6)	24.2 (3.8)	26.4 (4.3)	25.5 (4.8)	24.4 (3.7)	26.2 (4.3)	25.0 (4.0)	23.8 (3.4)
Missing BMI	3038 (0.7)	659 (0.6)	44 (0.6)	94 (0.7)	139 (0.4)	46 (0.9)	51 (0.9)	69 (0.7)	32 (0.6)	49 (0.6)
SBP, mean (SD), mm Hg^b^	124.5 (14.3)	121.5 (14.6)	124.6 (14.8)	119.4 (14.6)	124.2 (14.3)	123.8 (14.6)	119.7 (14.2)	120.2 (14.2)	120.5 (14.4)	118.5 (14.4)
DBP, mean (SD), mm Hg	73.8 (9.7)	73.1 (9.8)	74.6 (10.1)	71.7 (9.7)	73.9 (9.8)	73.3 (9.8)	72.6 (9.8)	72.6 (9.4)	72.6 (9.5)	72.2 (9.7)
TC, mean (SD), mg/dL^b^	198.2 (35.4)	195.8 (34.4)	195.3 (35.3)	194.9 (32.8)	195.7 (35.3)	199.2 (35.7)	196.2 (32.9)	190.9 (34.0)	198.0 (34.8)	197.3 (33.3)
LDL-C, mean (SD), mg/dL	118.4 (31.2)	114.4 (30.6)	114.3 (31.5)	113.5 (29.3)	113.6 (31.4)	113.3 (31.6)	113.8 (29.6)	113.9 (30.0)	116.4 (30.9)	116.0 (29.8)
Missing LDL-C	5264 (1.2)	1085 (1.0)	101 (1.3)	111 (0.8)	296 (0.8)	66 (1.3)	60 (1.0)	89 (0.9)	59 (1.1)	66 (0.8)
HDL-C, mean (SD), mg/dL^b^	53.2 (14.4)	54.1 (13.4)	52.2 (13.3)	55.7 (13.7)	53.8 (12.8)	57.9 (14.6)	54.4 (13.4)	49.6 (12.3)	55.5 (13.7)	53.9 (13.2)
eGFR, mL/min/1.73 m^2^^b^	86.7 (17.3)	94.5 (17.6)	92.2 (18.7)	93.6 (17.0)	93.3 (18.7)	90.0 (17.58)	97.0 (16.1)	94.2 (17.0)	94.3 (17.5)	98.1 (16.3)
Hemoglobin A_1c_, mg/dL	6.4 (1.2)	6.6 (1.2)	6.9 (1.6)	6.4 (1.0)	6.7 (1.2)	6.6 (1.1)	6.4 (1.0)	6.6 (1.1)	6.5 (1.1)	6.4 (1.1)
Missing hemoglobin A_1c_	324392 (76.5)	77954 (70.3)	5147 (66.7)	10795 (77.8)	22505 (63.4)	3518 (67.3)	4086 (69.9)	6593 (67.3)	3781 (72.8)	6728 (78.9)
UACR, mean (SD), mg/g	53.5 (326.5)	84.2 (374.3)	142.7 (615.7)	69.1 (315.9)	105.2 (419.9)	80.0 (370.0)	82.9 (370.4)	54.7 (332.0)	68.4 (286.3)	69.1 (250.6)
Missing UACR	347337 (81.9)	86492 (78.0)	5672 (73.5)	11499 (82.9)	25439 (71.7)	3904 (74.7)	4769 (81.6)	7507 (76.7)	4216 (81.1)	7282 (85.4)
SDI deciles										
1 (lowest level of social deprivation)	67644 (16.0)	16724 (15.1)	785 (10.2)	2989 (21.6)	3589 (10.1)	827 (15.8)	1407 (24.1)	2061 (21.1)	629 (12.1)	1109 (13.0)
2	58406 (13.8)	13289 (12.0)	760 (9.9)	1909 (13.8)	3895 (11.0)	725 (13.9)	806 (13.8)	1427 (14.6)	509 (9.8)	754 (8.8)
3	55844 (13.2)	12590 (11.4)	791 (10.3)	1791 (12.9)	3450 (9.7)	713 (13.7)	874 (15.0)	1249 (12.8)	502 (9.7)	857 (10.1)
4	51993 (12.3)	11993 (10.8)	829 (10.8)	1599 (11.5)	3715 (10.5)	665 (12.7)	647 (11.1)	1051 (10.7)	537 (10.3)	770 (9.0)
5	45944 (10.8)	11336 (10.2)	977 (12.7)	1243 (9.0)	4175 (11.8)	549 (10.5)	537 (9.2)	868 (8.9)	544 (10.5)	745 (8.7)
6	40526 (9.6)	9652 (8.7)	768 (10.0)	1021 (7.4)	3215 (9.1)	487 (9.3)	384 (6.6)	761 (7.8)	508 (9.8)	795 (9.3)
7	35350 (8.3)	11660 (10.5)	908 (11.8)	1305 (9.4)	4386 (12.4)	443 (8.5)	358 (6.1)	697 (7.1)	601 (11.6)	1083 (12.7)
8	34443 (8.1)	10115 (9.1)	821 (10.6)	964 (7.0)	3528 (9.9)	387 (7.4)	348 (6.0)	760 (7.8)	541 (10.4)	1218 (14.3)
9	20694 (4.9)	7284 (6.6)	613 (7.9)	635 (4.6)	2745 (7.7)	228 (4.4)	257 (4.4)	489 (5.0)	392 (7.5)	816 (9.6)
10 (highest level of social deprivation)	13167 (3.1)	6172 (5.6)	459 (6.0)	412 (3.0)	2785 (7.8)	199 (3.8)	220 (3.8)	427 (4.4)	430 (8.3)	374 (4.4)
Unknown	266 (0.1)	40 (0.04)	5 (0.1)	6 (0.04)	7 (0.02)	3 (0.1)	5 (0.1)	3 (0.03)	4 (0.1)	3 (0.04)
Current smoker	31303 (7.4)	4409 (4.0)	513 (6.6)	297 (2.1)	1647 (4.6)	249 (4.8)	241 (4.1)	291 (3.0)	151 (2.9)	345 (4.0)
Treated hypertension	127409 (30.0)	34140 (30.8)	2705 (35.1)	3493 (25.2)	14 537 (41.0)	1997 (38.2)	1646 (28.2)	2418 (24.7)	1372 (26.4)	1718 (20.2)
Diabetes	38747 (9.1)	14393 (13.0)	1289 (16.7)	1223 (8.8)	6127 (17.3)	796 (15.2)	670 (11.5)	1497 (15.3)	574 (11.0)	718 (8.4)
Statin use	118471 (27.9)	31898 (28.8)	2333 (30.2)	3254 (23.5)	12449 (35.1)	1970 (37.7)	1476 (25.3)	2702 (27.6)	1536 (29.6)	1910 (22.4)

Abbreviations: BMI, body mass index (calculated as weight in kilograms divided by height in meters squared); DBP, diastolic blood pressure; eGFR, estimated glomerular filtration rate; HDL-C, high-density lipoprotein cholesterol; LDL-C, low-density lipoprotein cholesterol; SBP, systolic blood pressure; SDI, Social Deprivation Index; TC, total cholesterol; UACR, urine albumin-creatinine ratio.

SI conversion factors: To convert cholesterol to millimoles per liter, multiply by 0.0259; hemoglobin A_1c_ to proportion of total hemoglobin, multiply by 0.01.

^a^ Unless otherwise indicated.

^b^ Individuals with missing values were excluded.

During a median (IQR) 10.0 (4.7-10.0) years of follow-up, 31 556 adults experienced a CVD event. Of those, 4135 were non-Hispanic Asian adults, 416 were non-Hispanic Native Hawaiian and Other Pacific Islander adults, and 27 005 were non-Hispanic White adults. The crude incidence rate of total CVD events was 4.70 per 1000 person-years in non-Hispanic Asian adults and 6.85 per 1000 person-years in non-Hispanic Native Hawaiian and Other Pacific Islander adults compared with 8.20 per 1000 person-years in non-Hispanic White adults (eTable 4 in Supplement 1). When adjusted for age and sex, non-Hispanic Native Hawaiian and Other Pacific Islander adults had the highest incident rate of total CVD events at 5.97 (95% CI, 5.42-6.57) per 1000 person-years compared with 3.73 (95% CI, 3.61-3.85) per 1000 person-years and 5.22 (95% CI, 5.13-5.31) per 1000 person-years for non-Hispanic Asian adults and non-Hispanic White adults, respectively (Table 2). Of these groups, non-Hispanic Native Hawaiian and Other Pacific Islander adults also had the highest adjusted incident rate of ASCVD (3.36 [95% CI, 2.95-3.83] per 1000 person-years) and HF (2.78 [95% CI, 2.43-3.17] per 1000 person-years). Among Asian ethnic groups, Filipino adults had the highest adjusted incidence rate of total CVD (4.09 [95% CI, 3.87-4.33] per 1000 person-years), ASCVD (2.46 [95% CI, 2.29-2.64] per 1000 person-years), and HF (1.73 [95% CI, 1.59-1.89] per 1000 person-years). South Asian adults had the highest incidence rate of MI (1.35 [95% CI, 1.13-1.61] per 1000 person-years), whereas Southeast Asian adults had the highest incidence rate of ischemic stroke (0.83 [95% CI, 0.62-1.10] per 1000 person-years).

**Table 2.** Age and Sex-Adjusted Incidence (95% CI) of Cardiovascular Disease Events Per 1000 Person-Years

Event	Non-Hispanic White (n = 424277)	Non-Hispanic Asian (n = 110855	Non-Hispanic Native Hawaiian and Other Pacific Islander (n = 7716)	Disaggregated non-Hispanic Asian ethnic groups (n = 83 947)						
				Chinese (n = 13874)	Filipino (n = 35490)	Japanese (n = 5226)	Korean (n = 5843)	South Asian (n = 9793)	Southeast Asian (n = 5197)	Vietnamese (n = 8524)
Total CVD	5.22 (5.13-5.31)	3.73 (3.61-3.85)	5.97 (5.42-6.57)	2.60 (2.37-2.86)	4.09 (3.87-4.33)	3.06 (2.72-3.44)	2.55 (2.22-2.93)	3.47 (3.12-3.86)	3.44 (2.99-3.95)	2.79 (2.45-3.18)
MI	1.53 (1.49-1.58)	1.15 (1.08-1.22)	1.97 (1.65-2.35)	0.58 (0.47-0.72)	1.32 (1.20-1.46)	0.95 (0.74-1.21)	0.74 (0.56-0.99)	1.35 (1.13-1.61)	1.01 (0.77-1.33)	0.81 (0.63-1.03)
Ischemic stroke	0.91 (0.87-0.95)	0.76 (0.71-0.82)	1.12 (0.9-1.39)	0.69 (0.57-0.83)	0.80 (0.71-0.91)	0.64 (0.50-0.83)	0.64 (0.49-0.84)	0.55 (0.42-0.72)	0.83 (0.62-1.10)	0.72 (0.56-0.93)
Heart failure^a^	2.40 (2.34-2.46)	1.41 (1.34-1.48)	2.52 (2.19-2.9)	0.88 (0.76-1.02)	1.57 (1.43-1.71)	1.06 (0.88-1.27)	0.67 (0.53-0.85)	1.31 (1.11-1.54)	1.17 (0.94-1.46)	0.78 (0.62-0.98)
ICH	0.62 (0.59-0.65)	0.76 (0.71-0.82)	0.96 (0.75-1.23)	0.57 (0.47-0.7)	0.65 (0.57-0.75)	0.61 (0.47-0.79)	0.71 (0.54-0.92)	0.43 (0.32-0.58)	0.61 (0.44-0.85)	0.69 (0.53-0.89)
CHD death	0.08 (0.07-0.09)	0.04 (0.03-0.05)	0.08 (0.04-0.16)	0.02 (0.01-0.04)	0.01 (0-0.02)	0.01 (0-0.03)	0.02 (0-0.05)	0 (0-0.03)	0.01 (0-0.06)	0 (NA)
Stroke death	0.01 (0.01-0.02)	0.01 (0-0.01)	0.01 (0-0.06)	0 (NA)	0 (0-0.02)	0.01 (0-0.05)	0.01 (0-0.07)	0 (NA)	0 (NA)	0 (NA)
ASCVD	2.74 (2.68-2.81)	2.19 (2.10-2.29)	3.36 (2.95-3.83)	1.48 (1.30-1.68)	2.46 (2.29-2.64)	1.86 (1.59-2.18)	1.57 (1.31-1.89)	2.10 (1.82-2.41)	2.13 (1.77-2.55)	1.71 (1.44-2.02)
Heart failure^b^	2.58 (2.52-2.64)	1.55 (1.48-1.62)	2.78 (2.43-3.17)	0.93 (0.81-1.07)	1.73 (1.59-1.89)	1.19 (1.00-1.40)	0.74 (0.59-0.93)	1.44 (1.24-1.68)	1.25 (1.01-1.55)	0.84 (0.67-1.04)

Abbreviations: ASCVD, atherosclerotic cardiovascular disease; CHD, coronary heart disease; CVD, cardiovascular disease; ICH, intracranial hemorrhage; MI, myocardial infarction; NA, not applicable.

^a^ Heart failure as one of the composite total CVD, censored at other CVD events.

^b^ Heart failure as a separate outcome.

**Figure 2.**
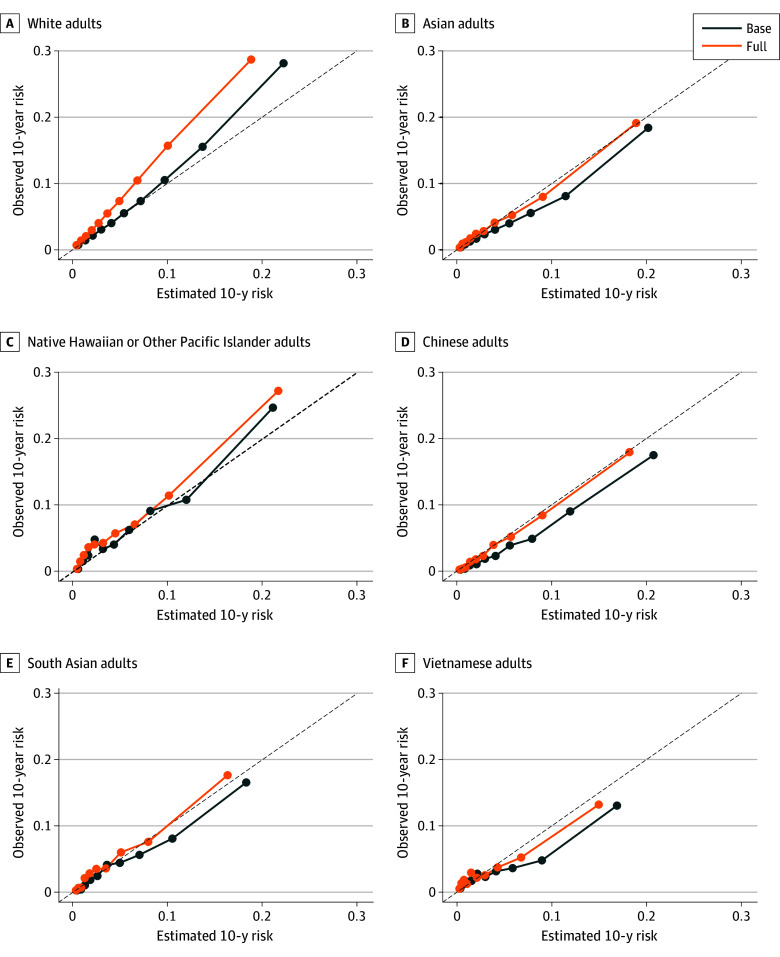
Calibration Curves of PREVENT Base and Full Models for Total Cardiovascular Outcome The calibration curve within each racial and ethnic group shows the mean predicted risk vs the observed event rate estimated from the Kaplan-Meier survival analysis by deciles of predicted risk.

For total CVD events, both the PREVENT base (Harrell C index, 0.764; 95% CI, 0.761-0.767) and full (Harrell C index, 0.767; 95% CI, 0.764-0.770) equations demonstrated good discrimination in non-Hispanic White adults (Table 3). Compared with non-Hispanic White adults, discrimination was marginally higher in non-Hispanic Asian adults in both the PREVENT base (Harrell C index, 0.773; 95% CI, 0.765-0.779; C index change, 0.009; 95% CI, 0.001-0.016) and full (Harrell C index, 0.776; 95% CI, 0.769-0.783; C index change, 0.009; 95% CI, 0.001-0.017) equations. In contrast, discrimination among non-Hispanic Native Hawaiian and Other Pacific Islander adults (Harrell C index, 0.757; 95% CI, 0.733-0.780 for the PREVENT base equation; C index change, −0.007; 95% CI, −0.032 to 0.015) was similar to that of non-Hispanic White adults. Among Asian ethnic groups, performance varied, with the highest discrimination observed in Chinese adults (Harrell C index, 0.806; 95% CI, 0.787-0.826) and the lowest in Vietnamese adults (Harrell C index, 0.738; 95% CI, 0.701-0.774). Both the PREVENT base and full equations underestimated total CVD risk in non-Hispanic Native Hawaiian and Other Pacific Islander (mean calibration, 0.86; 95% CI, 0.76-0.95) adults and non-Hispanic White (mean calibration, 0.84; 95% CI, 0.83-0.85) (Table 3, Figure 2). In contrast, the PREVENT base equation generally overestimated total CVD risk in non-Hispanic Asian (mean calibration, 1.18; 95% CI, 1.14-1.21) and Asian ethnic groups (mean calibration range, 1.04-1.39), whereas the PREVENT full equation underestimated it (mean calibration, 0.96; 95% CI,. 0.93-0.98) (Table 3 and Figure 2; eFigure 1 in Supplement 1).

**Table 3.** Harrell C Index and Mean Calibration Comparison (95% CI) for Total Cardiovascular Disease Applying PREVENT Base and Full Equations Applying Different Ethnic Groups

Measure	Non-Hispanic White (n = 424277)	Non-Hispanic Asian (n = 110855)	Non-Hispanic Native Hawaiian and Other Pacific Islander (n = 7716)	Disaggregated non-Hispanic Asian ethnic groups (n = 83 947)						
				Chinese (n = 13874)	Filipino (n = 35490)	Japanese (n = 5226)	Korean (n = 5843)	South Asian (n = 9793)	Southeast Asian (n = 5197)	Vietnamese (n = 8524)
**PREVENT base equation for total CVD outcome**										
Harrell C index	0.764 (0.761 to 0.767)	0.773 (0.765 to 0.779)	0.757 (0.733 to 0.780)	0.806 (0.787 to 0.826)	0.760 (0.746 to 0.773)	0.757 (0.727 to 0.786)	0.748 (0.714 to 0.783)	0.761 (0.732 to 0.791)	0.755 (0.721 to 0.790)	0.738 (0.701 to 0.774)
C index difference	0.0 [Reference]	0.009 (0.001 to 0.016)	−0.007 (−0.032 to 0.015)	0.042 (0.022 to 0.057)	−0.004 (−0.016 to 0.009)	−0.007 (−0.055 to 0.017)	−0.016 (−0.094 to 0.086)	−0.003 (−0.054 to 0.005)	−0.009 (−0.028 to 0.022)	−0.026 (−0.039 to 0.019)
Mean calibration	0.84 (0.83 to 0.85)	1.18 (1.14 to 1.21)	0.86 (0.76 to 0.95)	1.29 (1.19 to 1.40)	1.06 (1.02 to 1.11)	1.23 (1.12 to 1.37)	1.39 (1.24 to 1.56)	1.08 (0.97 to 1.18)	1.04 (0.91 to 1.19)	1.23 (1.08 to 1.38)
**PREVENT full equation for total CVD outcome**										
Harrell C index	0.767 (0.764 to 0.770)	0.776 (0.769 to 0.783)	0.765 (0.736 to 0.786)	0.810 (0.790 to 0.829)	0.764 (0.751 to 0.778)	0.761 (0.732 0.790)	0.745 (0.712 to 0.779)	0.769 (0.740 to 0.799)	0.760 (0.726 to 0.794)	0.735 (0.697 to 0.773)
C index difference	0.0 [Reference]	0.009 (0.001 to 0.017)	−0.002 (−0.028 to 0.020)	0.043 (0.025 to 0.056)	−0.003 (−0.014 to 0.011)	−0.006 (−0.058 to 0.011)	−0.022 (−0.121 to 0.071)	0.002 (−0.061 to 0.002)	−0.007 (−0.022 to 0.028)	−0.032 (−0.039 to 0.021)
Mean calibration	0.63 (0.62 to 0.64)	0.96 (0.93 to 0.98)	0.74 (0.66 to 0.82)	0.99 (0.91 to 1.07)	0.91 (0.88 to 0.96)	0.98 (0.89 to 1.09)	1.07 (0.96 to 1.20)	0.84 (0.76 to 0.93)	0.83 (0.72 to 0.95)	0.97 (0.85 to 1.09)

Abbreviation: CVD, cardiovascular disease.

For ASCVD events, discrimination was similar across non-Hispanic Asian, non-Hispanic Native Hawaiian and Other Pacific Islander, and non-Hispanic White adults for both PREVENT equations (eTable 5 in Supplement 1). Among Asian subgroups, the highest discrimination was observed in Chinese adults for both the PREVENT base and full equations (Harrell C index,0.798; 95% CI, 0.770-0.821 for the base equation; Harrell C index, 0.802; 95% CI, 0.774-0.823 for the full equation). Conversely, the lowest discrimination was observed in Korean (Harrell C index, 0.728; 95% CI, 0.689-0.773) adults for the PREVENT base equation and Vietnamese (Harrell C index, 0.721; 95% CI, 0.679-0.764) adults for the PREVENT full equation. The PREVENT base equation overestimated ASCVD risk in non-Hispanic Asian overall (mean calibration, 1.27; 95% CI, 1.22-1.32) and Asian subgroup populations of Chinese (mean calibration, 1.49; 95% CI, 1.34-1.68), Filipino (mean calibration, 1.13; 95% CI, 1.04-1.20), Japanese (mean calibration, 1.36; 95% CI, 1.20-1.55), Korean (mean calibration, 1.50; 95% CI, 1.23-1.82), and Vietnamese (mean calibration, 1.30; 95% CI, 1.11-1.49) (eTable 5 and eFigure 2 in Supplement 1). The PREVENT full equation was well calibrated for ASCVD risk in most Asian subgroup populations, although some overestimation remained in Chinese (mean calibration, 1.18; 95% CI, 1.07-1.33) adults.

For HF events, the PREVENT base and full equations demonstrated good discrimination in non-Hispanic Asian, non-Hispanic Native Hawaiian and Other Pacific Islander, and non-Hispanic White adults (eTable 5 in Supplement 1). Compared with non-Hispanic White adults (Harrell C index, 0.805; 95% CI, 0.801-0.808 for PREVENT base equation; Harrell C index, 0.808; 95% CI, 0.805-0.811 for PREVENT full equation), discrimination was higher in non-Hispanic Asian adults in both the PREVENT base (Harrell C index, 0.824; 95% CI, 0.815-0.833) and full (Harrell C index, 0.831; 95% CI, 0.822-0.839) equations. The PREVENT base equation overestimated HF risk across all Asian subgroups, whereas the PREVENT full equation overestimated HF risk only in Chinese (mean calibration, 1.22; 95% CI, 1.10-1.38), Korean (mean calibration, 1.55; 95% CI, 1.24-2.02), and Vietnamese (mean calibration, 1.42; 95% CI, 1.17-1.78) adults (eTable 5 and eFigure 3 in Supplement 1).

## Discussion

In this study, the PREVENT equations demonstrated overall good discrimination and calibration for aggregate non-Hispanic Asian and non-Hispanic Native Hawaiian and Other Pacific Islander populations across outcomes of total CVD, ASCVD, and HF. For total CVD and HF outcomes, discrimination was marginally higher among non-Hispanic Asian compared with non-Hispanic White adults. However, the PREVENT base and full equations, with the exception of the PREVENT full equation for total CVD, generally overestimated total CVD, ASCVD, and HF risk among non-Hispanic Asian adults, whereas the PREVENT base and full equations underestimated the risk among non-Hispanic Native Hawaiian and Other Pacific Islander and non-Hispanic White populations. Model performance varied among disaggregated non-Hispanic Asian populations, underscoring the notion that risk prediction models may not be uniformly applicable across all disaggregated racial and ethnic groups.

Previous studies have shown that traditional risk calculators, including the pooled cohort equations, which were derived using only and non-Hispanic Black and White adults, may not fully account for racial and ethnic differences in CVD burden, particularly among Asian and Native Hawaiian and Other Pacific Islander subgroups with distinct risk profiles.^19,20^ Specifically, Filipino, Native Hawaiian and Other Pacific Islander, and South Asian adults have the highest prevalence of CVD events, likely due to a higher prevalence of diabetes, central adiposity, and other cardiometabolic risk factors.^12,21,22,23^ In contrast, Chinese adults generally exhibit lower rates of hypertension, hyperlipidemia, and diabetes, resulting in a comparatively lower CVD burden.^13,24^

Our study cohort reflected similar patterns of CVD risk factors and burden as reported in the literature. Non-Hispanic Filipino, Native Hawaiian and Other Pacific Islander, and South Asian adults had the highest mean BMIs, as well as higher prevalence of hypertension, diabetes, and higher CVD incidence rates. After adjusting for age and sex, our study found that Filipino adults had the highest incidence rate of total CVD, ASCVD, and HF among non-Hispanic Asian ethnic groups.

The PREVENT base and full equations demonstrated comparable discrimination performance in non-Hispanic Asian, non-Hispanic Native Hawaiian and Other Pacific Islander, and non-Hispanic White adults across all outcomes. For total CVD and HF outcomes, discrimination was even slightly higher among non-Hispanic Asian compared with non-Hispanic White adults. When further stratified by individual Asian ethnic groups, discrimination was highest in Chinese adults and lowest in Vietnamese adults, although the differences were modest.

Calibration results varied across non-Hispanic Asian, non-Hispanic Native Hawaiian and Other Pacific Islander, and non-Hispanic White adults. The PREVENT base and full equations generally overestimated total CVD, ASCVD, and HF risk, whereas the equations underestimated the risk among non-Hispanic Native Hawaiian and Other Pacific Islander and non-Hispanic White populations. Although the magnitude of miscalibration was modest, understanding model performance across racial and ethnic groups is important. Among non-Hispanic Asian adults, overestimation was reduced when applying the PREVENT full model. The improved calibration observed with the PREVENT full equation compared with the base equation among non-Hispanic Asian adults suggests that accounting for additional risk factors, including hemoglobin A_1c_, UACR, and area-level social deprivation via the SDI, may enhance model performance. However, the calibration of the PREVENT full equation was worse compared with the base equation among both non-Hispanic Native Hawaiian and Other Pacific Islander and non-Hispanic White adults. These varied results when applying the PREVENT base and full equations across racial and ethnic groups warrant further investigation. A higher proportion of missing values in hemoglobin A_1c_ and UACR among non-Hispanic Asian adults compared with non-Hispanic White adults may partly explain the differential impact.

Model performance varied among disaggregated non-Hispanic Asian populations. These findings suggest that although the PREVENT equations perform well when evaluating non-Hispanic Asians as a collective group, there remains variation in total CVD risk across individual groups that is not fully captured by the PREVENT model. Although the PREVENT full model incorporated area-level social deprivation, unmeasured individual-level social determinants of health may also influence model performance. A previous study demonstrated that including individual-level social determinants of health into the PREVENT plus SDI equation improved calibration in Black and White participants.^25^ Social determinants of health have been recognized as important upstream determinants of cardiovascular health and CVD outcomes across all Asian adults.^26,27^ However, their influence is not felt uniformly within the non-Hispanic Asian population. For example, median annual household income, which is a primary driver of socioeconomic position that helps determine an individual’s access to health care resources, differs widely between subgroups, ranging from approximately $44 000 per year (Burmese Americans) to $119 000 per year (Indian Americans).^27^ Similarly, food insecurity, which is associated with obesity in women, diabetes, and cardiovascular mortality, varies across Asian American subgroups, with higher prevalence often observed in Asian Indian, Filipino, Korean, and Vietnamese communities.^28,29,30,31^ Future studies are needed to assess whether incorporating individual-level social determinants of health improves CVD risk prediction across various Asian ethnic subgroups.

The current study found notable differences in calibration between non-Hispanic Asian and non-Hispanic Native Hawaiian and Other Pacific Islander adults when applying the PREVENT equations. These findings suggest that the consolidation of both groups into a single race category may obscure meaningful differences in risk prediction accuracy. Non-Hispanic Asian and non-Hispanic Native Hawaiian and Other Pacific Islander adults have been traditionally grouped together due to their relatively small population sizes and the historical categorization practices of federal datasets rather than scientific or clinical rationale.^32,33^ However, this practice not only diminishes the visibility of non-Hispanic Native Hawaiian and Other Pacific Islander adults in CVD, ASCVD, and HF research but also risks perpetuating systemic neglect of their unique social, cultural, and health characteristics.

Taken together, the results of this study highlight the ongoing need to refine total CVD, ASCVD, and HF risk prediction models to ensure equitable performance across diverse racial and ethnic populations. Future model development efforts should prioritize the inclusion of disaggregated racial and ethnic subgroup data to better reflect the heterogeneity of cardiovascular risk across populations.

### Limitations

This study has limitations, some of which are inherent to the study design. As with any retrospective cohort study, this study is susceptible to biases related to the quality of the historical data gathered. Moreover, we excluded individuals with missing or out-of-range values for key risk factors necessary for the PREVENT base equation. This approach excluded individuals who were younger and had lower rates of treated hypertension and statin use, suggesting systematic differences compared with those included. Due to these systemic differences, we did not perform multiple imputations for missing values. However, because our primary objective was to evaluate the performance of the PREVENT prediction model among different racial and ethnic groups, the potential for bias introduced by these exclusions is likely minimal. Although we applied both the PREVENT base and full equations, a substantial proportion of data for hemoglobin A_1c_ and UACR was missing because these tests were not routinely performed. The missing values may have affected the performance of the model. The cohort consists only of insured individuals enrolled within KPSC and thus may not accurately reflect non-Hispanic Asian and non-Hispanic Native Hawaiian and Other Pacific Islander adult populations; as a result, although KPSC provides health care to 4.9 million members (20% of the population in Southern California), the generalizability of these results to populations outside this health care system may be limited.

## Conclusions

In this retrospective cohort study of 542 848 KPSC adult members without baseline CVD, subgroup-specific differences in PREVENT base and full equation risk prediction were observed across disaggregated Asian and Native Hawaiian and Other Pacific Islander populations despite the equations’ overall strong performance. These findings underscore the importance of recognizing heterogeneity within Asian and Native Hawaiian and Other Pacific Islander populations when applying risk prediction models and highlight the ongoing need for tailored total CVD, ASCVD, and HF risk prediction models that account for the diverse nature of racial and ethnic health disparities.

## Notes

Supplement 1. **eFigure 1.** Calibration Plots of PREVENT Base and Full Models for Total Cardiovascular Disease Outcome for Filipino, Japanese, Korean, and South Asian Adults **eFigure 2.** Calibration Plots of PREVENT Base and Full Models for Atherosclerotic Cardiovascular Disease Outcome **eFigure 3.** Calibration Plots of PREVENT Base and Full Models for Heart Failure Outcome **eTable 1.** Characteristics of Missing Key Risk Factors or Out-of-Range **eTable 2.** Diagnosis Codes for Total Cardiovascular Disease Definitions **eTable 3.** Characteristics of Non-Hispanic Asian Population Who Reported Ethnicity versus Did Not **eTable 4.** Crude Incidence of Cardiovascular Events per 1,000 person-years **eTable 5.** Harrell’s C-index and Mean Calibration for Atherosclerotic Cardiovascular Disease (ASCVD) and Heart Failure (HF) Outcomes applying PREVENT Base and Full Equations by Different Ethnic Groups

Supplement 2.
Data Sharing Statement
