# Supplementary material for: PREVENT Equation Performance in Asian and Native Hawaiian and Other Pacific Islander Groups
Source: JAMA Netw Open. 2026 Feb 12;9(2):e2556915. doi: 10.1001/jamanetworkopen.2025.56915 (PMC12902880; doi:10.1001/jamanetworkopen.2025.56915)
Supplement: Supplement 1. — eFigure 1. Calibration Plots of PREVENT Base and Full Models for Total Cardiovascular Disease Outcome for Filipino, Japanese, Korean, and South Asian Adults eFigure 2. Calibration Plots of PREVENT Base and Full Models for Atherosclerotic Cardiovascular Disease Outcome eFigure 3. Calibration Plots of PREVENT Base and Full Models for Heart Failure Outcome eTable 1. Characteristics of Missing Key Risk Factors or Out-of-Range eTable 2. Diagnosis Codes for Total Cardiovascular Disease Definitions eTable 3. Characteristics of Non-Hispanic Asian Population Who Reported Ethnicity versus Did Not eTable 4. Crude Incidence of Cardiovascular Events per 1,000 person-years eTable 5. Harrell’s C-index and Mean Calibration for Atherosclerotic Cardiovascular Disease (ASCVD) and Heart Failure (HF) Outcomes applying PREVENT Base and Full Equations by Different Ethnic Groups [file jamanetwopen-e2556915-s001.pdf]

# Supplemental Online Content

Au M, Zhang Y, Zhou MM, et al. PREVENT equation performance in Asian and Native Hawaiian and Other Pacific Islander groups. *JAMA Netw Open*. 2026;9(2):e2556915. doi:10.1001/jamanetworkopen.2025.56915

**eFigure 1.** Calibration Plots of PREVENT Base and Full Models for Total Cardiovascular Disease Outcome for Filipino, Japanese, Korean, and South Asian Adults

**eFigure 2.** Calibration Plots of PREVENT Base and Full Models for Atherosclerotic Cardiovascular Disease Outcome

**eFigure 3.** Calibration Plots of PREVENT Base and Full Models for Heart Failure Outcome

**eTable 1.** Characteristics of Missing Key Risk Factors or Out-of-Range

**eTable 2.** Diagnosis Codes for Total Cardiovascular Disease Definitions

**eTable 3.** Characteristics of Non-Hispanic Asian Population Who Reported Ethnicity versus Did Not

**eTable 4.** Crude Incidence of Cardiovascular Events per 1,000 person-years

**eTable 5.** Harrell's C-index and Mean Calibration for Atherosclerotic Cardiovascular Disease (ASCVD) and Heart Failure (HF) Outcomes applying PREVENT Base and Full Equations by Different Ethnic Groups

This supplemental material has been provided by the authors to give readers additional information about their work.

© 2026 Au M et al. *JAMA Network Open.*

**eFigure 1. Calibration Plots of PREVENT Base and Full Models for Total Cardiovascular Disease Outcome for Filipino, Japanese, Korean, and South Asian Adults**

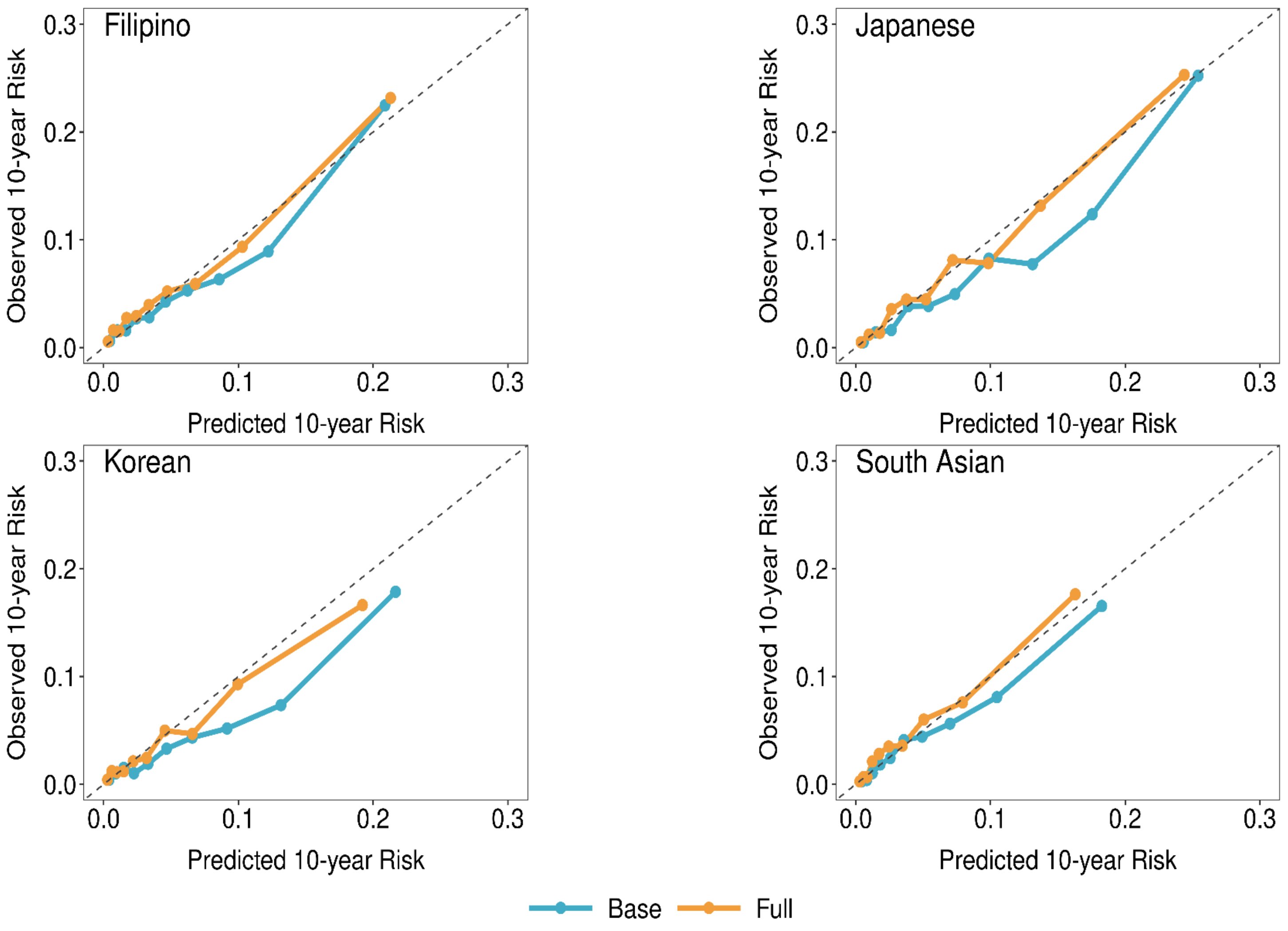


© 2026 Au M et al. *JAMA Network Open.*

**eFigure 2. Calibration Plots of PREVENT Base and Full Models for Atherosclerotic Cardiovascular Disease Outcome**

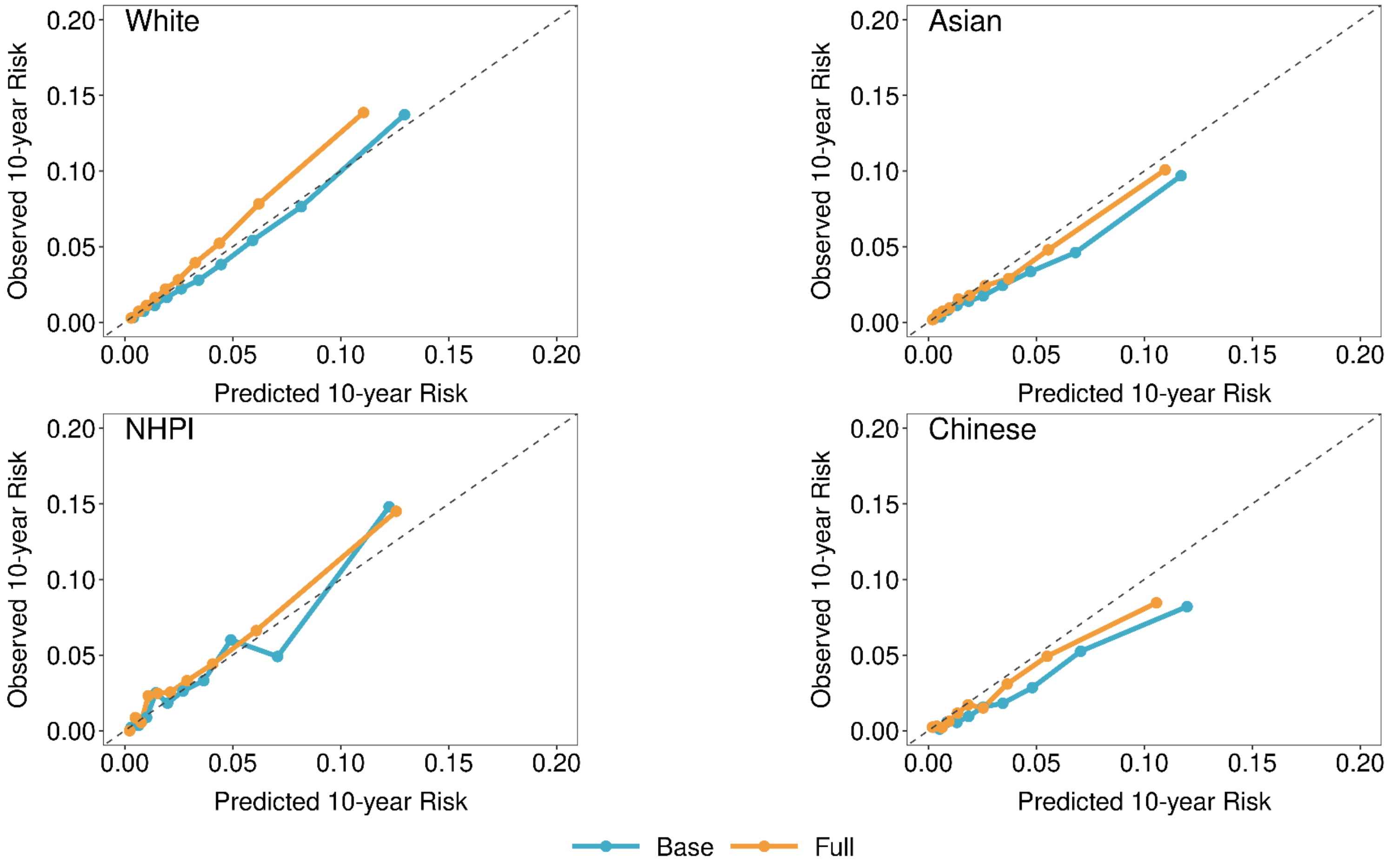


© 2026 Au M et al. *JAMA Network Open.*

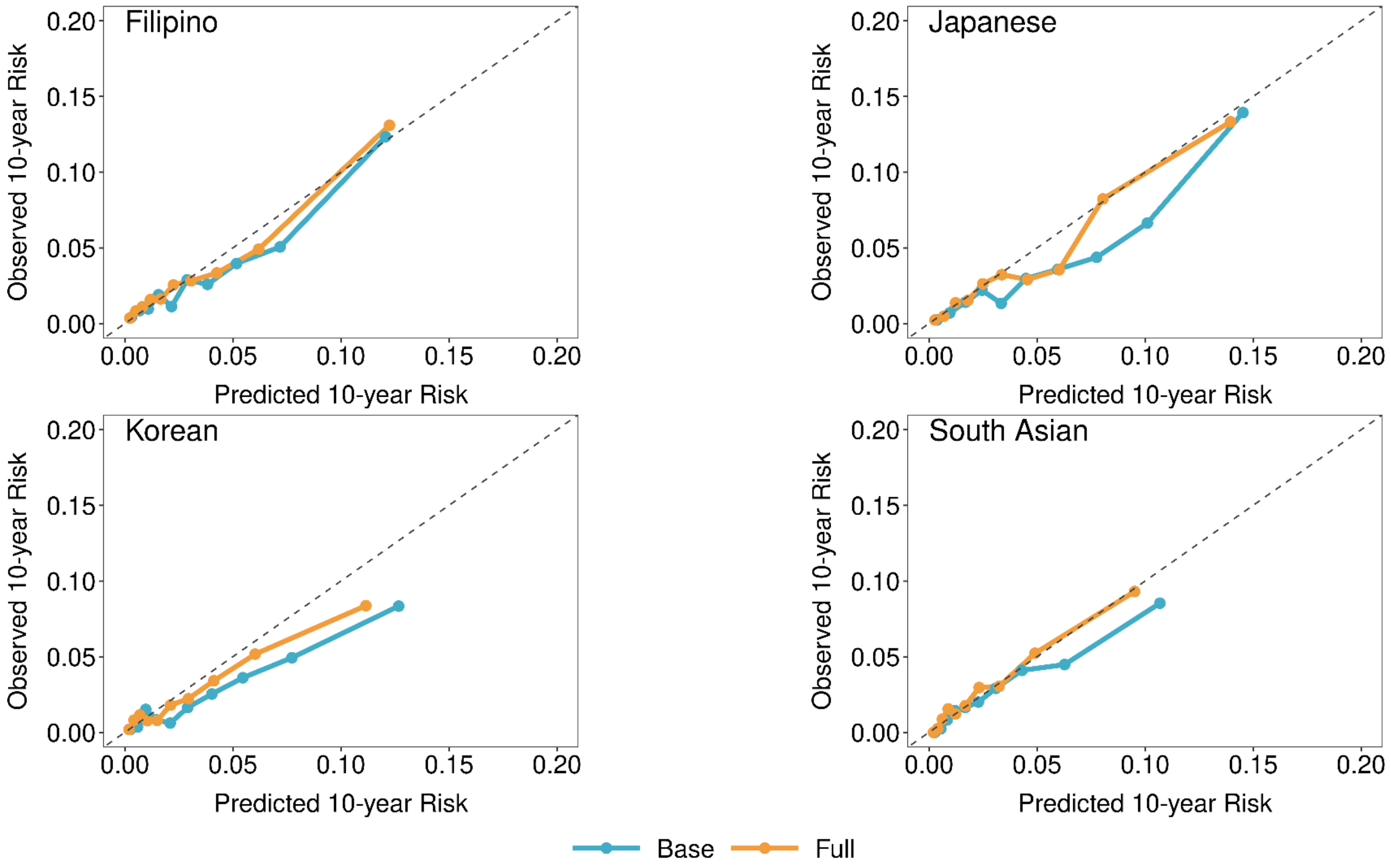


© 2026 Au M et al. *JAMA Network Open.*

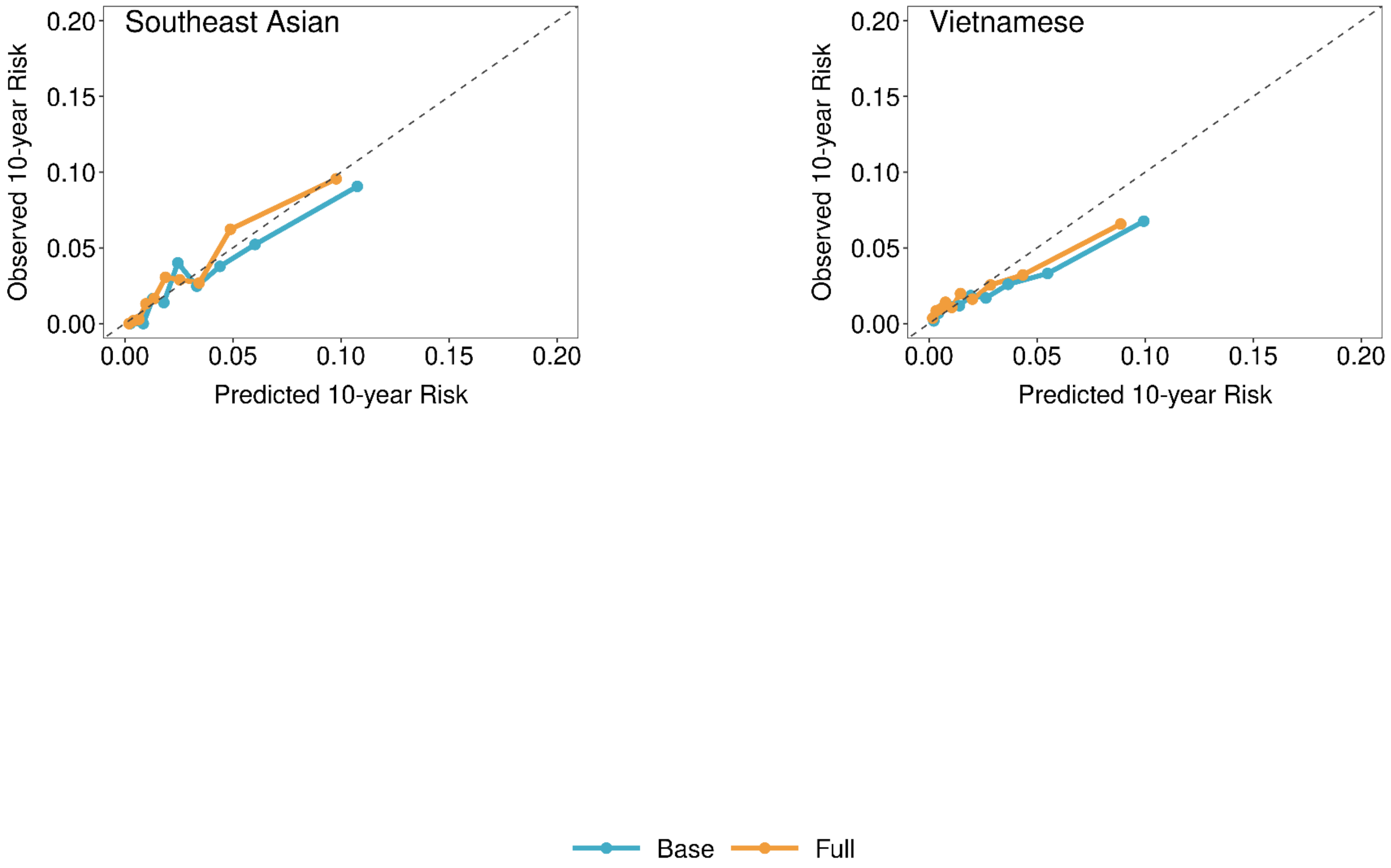


© 2026 Au M et al. *JAMA Network Open.*

**eFigure 3. Calibration Plots of PREVENT Base and Full Models for Heart Failure Outcome**

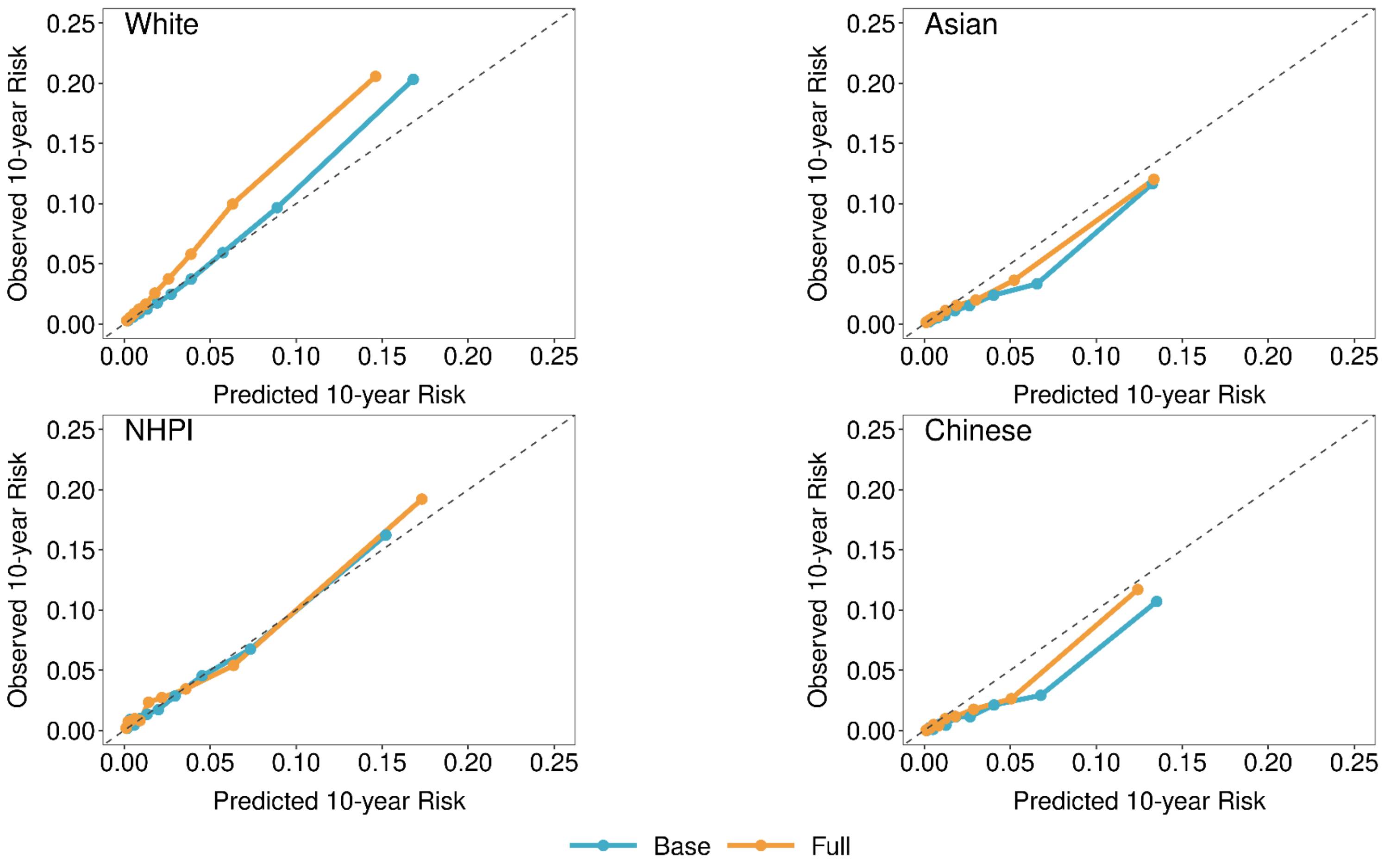


© 2026 Au M et al. *JAMA Network Open.*

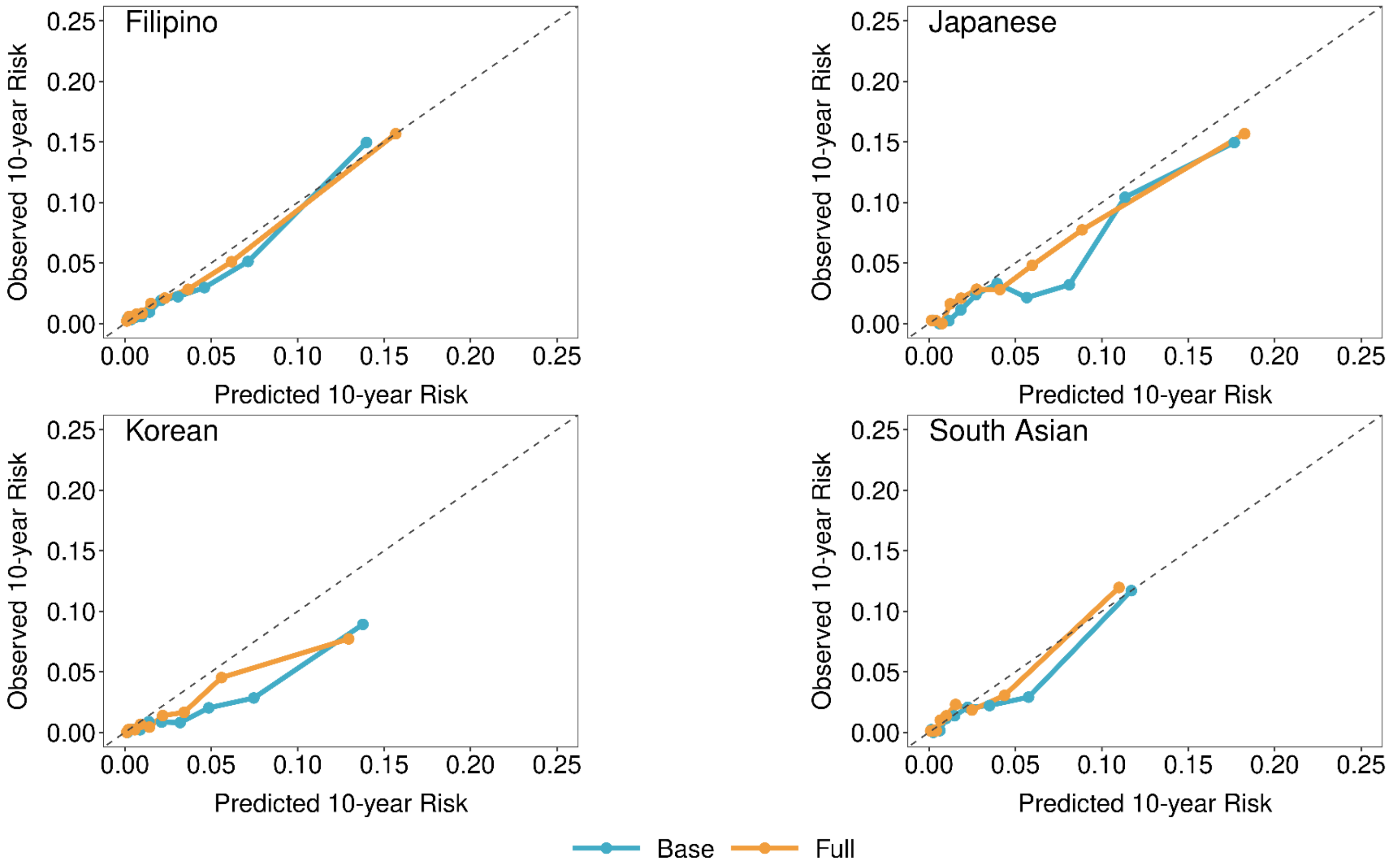


© 2026 Au M et al. *JAMA Network Open.*

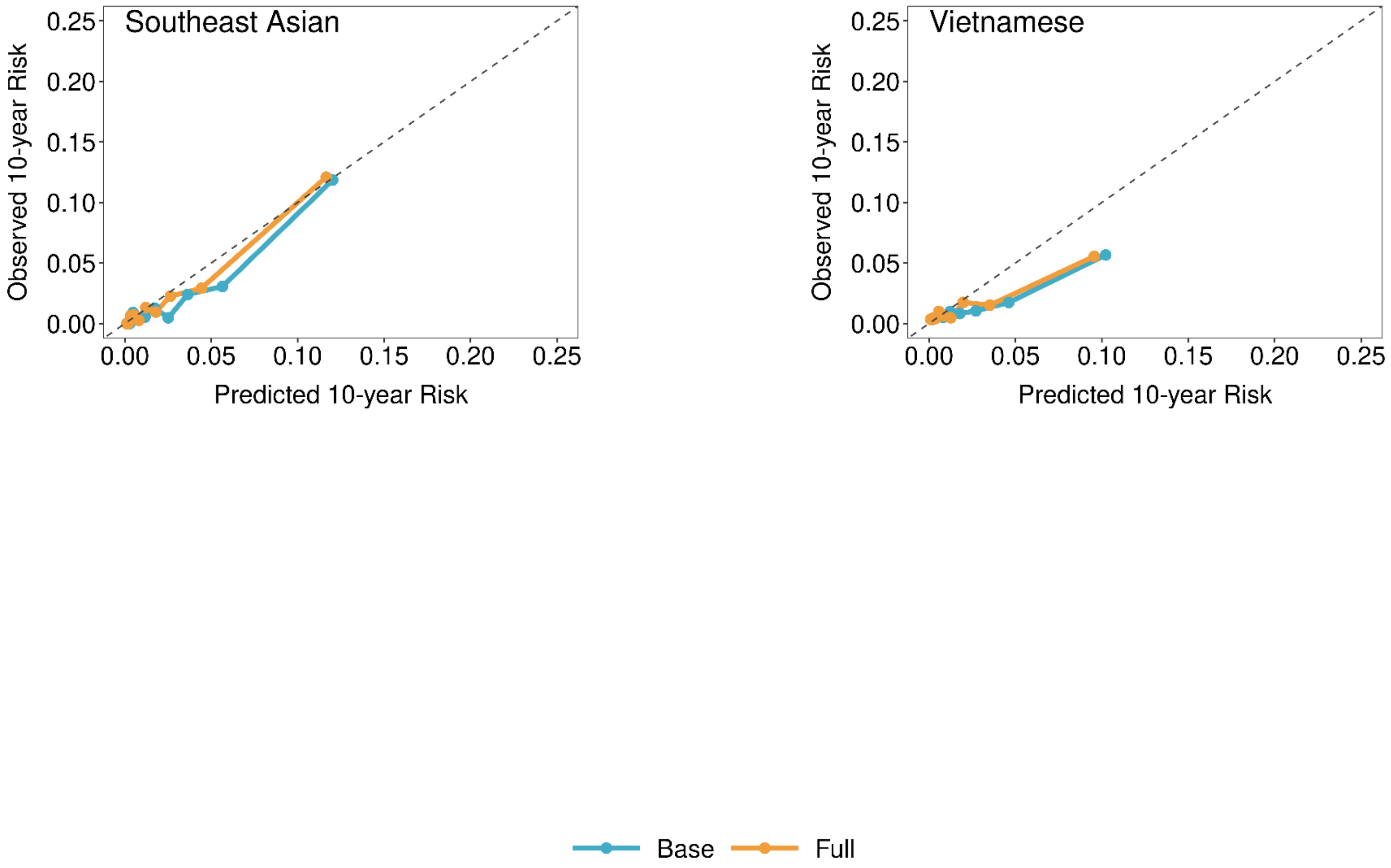


© 2026 Au M et al. *JAMA Network Open.*

**eTable 1. Characteristics of Missing Key Risk Factors or Out-of-Range**

| Characteristic | Missing Risk Factors or Out-of-Range (N = 496,430) |
|---|---|
| Age, years | 45.7 (11.2) |
| Female Sex | 45.6% |
| BMI, kg/m | 28.5 (6.1) |
| Missing BMI | 33.6% |
| SBP, mmHg | 121.2 (15.9) |
| Missing SBP | 40.1% |
| DBP, mmHg | 73.4 (10.6) |
| Missing DBP | 40.1% |
| TC, mg/dL | 189.6 (55.7) |
| Missing TC | 61.8% |
| LDL Cholesterol, mg/dL | 112.2 (43.7) |
| Missing LDL Cholesterol | 62.3% |
| HDL Cholesterol, mg/dL | 51.5 (17.9) |
| Missing HDL Cholesterol | 60.8% |
| eGFR, mL/min/1.73m² | 93.9 (21.0) |
| Missing eGFR | 65.1% |
| A1c, mg/dL | 6.8 (1.6) |
| Missing A1c | 92.6% |
| UACR, mg/g | 203.3 (1844.1) |
| Missing UACR | 94.3% |
| SDI deciles | |
| 1 (lowest level of social deprivation) | 10.5% |
| 2 | 10.2% |
| 3 | 9.4% |

© 2026 Au M et al. *JAMA Network Open.*

| | |
|---|---|
| 4 | 10.3% |
| 5 | 9.4% |
| 6 | 10.7% |
| 7 | 8.9% |
| 8 | 10.6% |
| 9 | 9.3% |
| 10 (highest level of social deprivation) | 9.8% |
| Unknown | 0.9% |
| Current Smoker | 5.3% |
| Treated Hypertension | 6.4% |
| Diabetes | 4.1% |
| Statin Use | 6.2% |

© 2026 Au M et al. *JAMA Network Open.*

**eTable 2. Diagnosis Codes for Total Cardiovascular Disease Definitions**

| Outcome | | | ICD Codes | Source | Principal diagnosis | Positive Predictive Values | Reference |
|---|---|---|---|---|---|---|---|
| Total CVD | ASCVD | Ischemic stroke<br><br>Intracerebral hemorrhage | ICD-9 433.x1, 434.x1, 436, and ICD-10 I63.x, G46.3, G46.4<br><br>ICD-9 430, 431, 432, 432.0, 432.1, 432.9, and ICD-10 I60.x, I61.x, I62.x | Inpatient files<br><br>Inpatient files | Y<br><br>Y or N | 80%-93% | Shirley AM, Morrisette KL, Choi SK, et al. Validation of ICD-10 hospital discharge diagnosis codes to identify incident and recurrent ischemic stroke from a US integrated healthcare system. Pharmacoepidemiol Drug Saf. 2023 Dec;32(12):1439-1445. doi: 10.1002/pds.5675. Epub 2023 Aug 1. PMID: 37528669; PMCID: PMC10830879.<br>Go AS, Hylek EM, Chang Y, et al. Anticoagulation therapy for stroke prevention in atrial fibrillation: how well do randomized trials translate into clinical practice? JAMA. Nov 26 2003;290(20):2685-92. doi:10.1001/jama.290.20.2685<br>Singer DE, Chang Y, Borowsky LH, et al. A new risk scheme to predict ischemic stroke and other thromboembolism in atrial fibrillation: the ATRIA study stroke risk score. J Am Heart Assoc. Jun 21 2013;2(3):e000250. doi:10.1161/JAHA.113.000250 |
| | | Non-fatal myocardial infarction | ICD-9 410.x0, 410.x1, 410.0-410.9 and ICD-10 I21.x | Inpatient files | Y | 91.2% | Reynolds K, Go AS, Leong TK, et al. Trends in Incidence of Hospitalized Acute Myocardial Infarction in the Cardiovascular Research Network (CVRN). *Am J Med*. Mar 2017;130(3):317-327. doi:10.1016/j.amjmed.2016.09.014 |
| | | Coronary Heart Disease Death | ICD-10 I20.x-I25.x (Kaiser Permanente Mortality Data has ICD-10 Only) | National death index and Kaiser Permanente sources | Not applicable | 75%-100% | Chung CP, Murray KT, Stein CM, Hall K, Ray WA. A computer case definition for sudden cardiac death. Pharmacoepidemiol Drug Saf 2010;19:563-72<br>Zaroff JG, Cheetham TC, Palmetto N, et al. Association of Azithromycin Use With Cardiovascular Mortality. *JAMA Netw Open*. |

© 2026 Au M et al. *JAMA Network Open.*

| | | | | | | | |
|---|---|---|---|---|---|---|---|
| | | | | | | | Jun 1 2020;3(6):e208199. doi:10.1001/jamanetworkopen.2020.8199 |
| | | Stroke Death | ICD-10 I60.x-I69.x (Kaiser Permanente Mortality Data has ICD-10 Only) | National death index and Kaiser Permanente sources | Not applicable | | |
| | | Heart Failure | ICD-9 428.x, and ICD-10 I50.x | Inpatient files | Y or N | >95% | Gurwitz JH, Magid DJ, Smith DH, et al. Contemporary prevalence and correlates of incident heart failure with preserved ejection fraction. *Am J Med*. May 2013;126(5):393-400. doi:10.1016/j.amjmed.2012.10.022<br>McKee PA, Castelli WP, McNamara PM, Kannel WB. The natural history of congestive heart failure: the Framingham study. N Engl J Med. Dec 23 1971;285(26):1441-6. doi:10.1056/NEJM197112232852601<br>Go AS, Yang J, Ackerson LM, Lepper K, Robbins S, Massie BM, Shlipak MG. Hemoglobin level, chronic kidney disease, and the risks of death and hospitalization in adults with chronic heart failure: the Anemia in Chronic Heart Failure: Outcomes and Resource Utilization (ANCHOR) Study. Circulation. Jun 13 2006;113(23):2713-23. doi:10.1161/CIRCULATIONAHA.105.577577 |

© 2026 Au M et al. *JAMA Network Open.*

**eTable 3. Characteristics of the Non-Hispanic Asian Population by Ethnicity Reporting Status**

| Characteristic | Non-Hispanic Asian Overall (N=110,855) | Asian Who Reported Ethnicity (N=83,947) | Asian Who Did Not Report Ethnicity (N=26,908) |
|---|---|---|---|
| Age, years | 52.5 (11.9) | 53.1 (11.9) | 50.8 (11.8) |
| Female Sex | 59.8% | 59.9% | 59.4% |
| BMI, kg/m² | 25.5 (4.3) | 25.5 (4.2) | 25.6 (4.6) |
| SBP, mmHg | 121.5 (14.6) | 121.8 (14.6) | 120.8 (14.5) |
| DBP, mmHg | 73.1 (9.8) | 73.0 (9.7) | 73.1 (9.9) |
| TC, mg/dL | 195.8 (34.4) | 195.6 (34.4) | 196.5 (34.5) |
| LDL Cholesterol, mg/dL | 114.4 (30.6) | 114.0 (30.6) | 115.3 (30.5) |
| HDL Cholesterol, mg/dL | 54.1 (13.4) | 54.0 (13.3) | 54.2 (13.5) |
| eGFR, mL/min/1.73m² | 94.5 (17.6) | 94.0 (17.8) | 96.1 (17.1) |
| A1c, mg/dL | 6.6 (1.2) | 6.6 (1.2) | 6.5 (1.2) |
| UACR, mg/g | 84.2 (374.3) | 87.6 (377.5) | 71.3 (361.7) |
| SDI deciles | | | |
| 1 (lowest level of social deprivation) | 15.1% | 15.0% | 15.3% |
| 2 | 12.0% | 11.9% | 12.1% |
| 3 | 11.4% | 11.2% | 11.7% |
| 4 | 10.8% | 10.7% | 11.2% |
| 5 | 10.2% | 10.3% | 9.9% |
| 6 | 8.7% | 8.5% | 9.2% |
| 7 | 10.5% | 10.6% | 10.4% |
| 8 | 9.1% | 9.2% | 8.8% |
| 9 | 6.6% | 6.6% | 6.4% |
| 10 (highest level of social deprivation) | 5.6% | 5.8% | 4.9% |
| Current Smoker | 4.0% | 3.8% | 4.4% |
| Treated Hypertension | 30.8% | 32.4% | 25.9% |

© 2026 Au M et al. *JAMA Network Open.*

| | | | |
|---|---|---|---|
| Diabetes | 13.0% | 13.8% | 10.4% |
| Statin Use | 28.8% | 30.1% | 24.5% |

The data is reported as mean (SD) or %.
Abbreviations: NH = non-Hispanic; NHPI = Native Hawaiian or Pacific Islander; SDI = Social deprivation index; SE = South East; BMI = body mass index; SBP = systolic blood pressure; DBP = diastolic blood pressure; TC = total cholesterol; LDL = low-density lipoprotein; HDL = high-density lipoprotein; eGFR = estimated glomerular filtration rate

© 2026 Au M et al. *JAMA Network Open.*

**eTable 4. Crude Incidence of Cardiovascular Events per 1,000 person-years**

| Event | NH White (N = 424,277) | NH Asian (N = 110,855 | NHPI (N = 7,716) | Disaggregated NH Asian Ethnic Groups (N=83,947) | | | | | | |
|---|---|---|---|---|---|---|---|---|---|---|
| | | | | Chinese (N = 13,874) | Filipino (N = 35,490) | Japanese (N = 5,226) | Korean (N = 5,843) | South Asian (N = 9,793) | SE Asian (N = 5,197) | Vietnamese (N = 8,524) |
| Total CVD | 8.20 (8.20, 8.20) | 4.70 (4.69, 4.71) | 6.85 (6.82, 6.88) | 4.35 (4.33, 4.37) | 5.74 (5.73, 5.75) | 7.10 (7.06, 7.14) | 4.54 (4.51, 4.57) | 4.63 (4.61, 4.65) | 4.90 (4.87, 4.93) | 3.52 (3.50, 3.54) |
| MI | 1.98 (1.98, 1.98) | 1.24 (1.24, 1.24) | 2.01 (1.99, 2.03) | 0.79 (0.78, 0.80) | 1.58 (1.57, 1.59) | 1.54 (1.52, 1.56) | 1.05 (1.04, 1.06) | 1.69 (1.68, 1.70) | 1.27 (1.25, 1.29) | 0.95 (0.94, 0.96) |
| Ischemic Stroke | 1.52 (1.52, 1.52) | 1.02 (1.02, 1.02) | 1.35 (1.34, 1.36) | 1.18 (1.17, 1.19) | 1.17 (1.16, 1.18) | 1.58 (1.56, 1.60) | 1.17 (1.16, 1.18) | 0.73 (0.72, 0.74) | 1.19 (1.17, 1.21) | 0.91 (0.90, 0.92) |
| Heart Failure | 4.35 (4.35, 4.35) | 2.01 (2.01, 2.01) | 3.23 (3.21, 3.25) | 1.83 (1.82, 1.84) | 2.65 (2.64, 2.66) | 3.30 (3.27, 3.33) | 1.51 (1.49, 1.53) | 2.05 (2.04, 2.06) | 1.98 (1.96, 2.00) | 1.15 (1.14, 1.16) |
| ICH | 0.89 (0.89, 0.89) | 0.90 (0.90, 0.90) | 1.04 (1.03, 1.05) | 0.98 (0.97, 0.99) | 0.94 (0.93, 0.95) | 1.47 (1.45, 1.49) | 1.30 (1.29, 1.31) | 0.58 (0.57, 0.59) | 0.88 (0.87, 0.89) | 0.88 (0.87, 0.89) |
| CHD Death | 0.17 (0.17, 0.17) | 0.06 (0.06, 0.06) | 0.12 (0.12, 0.12) | 0.08 (0.08, 0.08) | 0.04 (0.04, 0.04) | 0.09 (0.09, 0.09) | 0.08 (0.08, 0.08) | 0.01 (0.01, 0.01) | 0.05 (0.05, 0.05) | 0 (N/A) |

Abbreviations: NH = non-Hispanic; NHPI = Native Hawaiian or Pacific Islander; SE = South East; CVD = cardiovascular disease; MI = myocardial Infarction; ICH = intracranial hemorrhage; CHD = coronary heart disease.

© 2026 Au M et al. *JAMA Network Open.*

**eTable 5. Harrell's C-index and Mean Calibration for Atherosclerotic Cardiovascular Disease (ASCVD) and Heart Failure (HF) Outcomes applying PREVENT Base and Full Equations by Different Ethnic Groups**

| | **NH White (N = 424,277)** | **NH Asian (N = 110,855** | **NH NHPI (N = 7,716)** | **Disaggregated NH Asian Subgroups (N=83,947)** | | | | | | |
|---|---|---|---|---|---|---|---|---|---|---|
| | | | | **Chinese (N = 13,874)** | **Filipino (N = 35,490)** | **Japanese (N = 5,226)** | **Korean (N = 5,843)** | **South Asian (N = 9,793)** | **SE Asian (N = 5,197)** | **Vietnamese (N = 8,524)** |
| **PREVENT Base Equation – ASCVD Outcome** | | | | | | | | | | |
| Harrell's C-index (95% CI) | 0.755 (0.751, 0.758) | 0.761 (0.752, 0.770) | 0.776 (0.744, 0.807) | 0.798 (0.770, 0.821) | 0.747 (0.727, 0.765) | 0.748 (0.718, 0.788) | 0.728 (0.689, 0.773) | 0.744 (0.695, 0.777) | 0.743 (0.712, 0.775) | 0.729 (0.689, 0.771) |
| Mean Calibration (95% CI) | 1.03 (1.01, 1.05) | 1.27 (1.22, 1.32) | 0.95 (0.85, 1.08) | 1.49 (1.34, 1.68) | 1.13 (1.04, 1.20) | 1.36 (1.20, 1.55) | 1.50 (1.23, 1.82) | 1.14 (0.98, 1.32) | 1.09 (0.90, 1.33) | 1.30 (1.11, 1.49) |
| **PREVENT Full Equation – ASCVD Outcome** | | | | | | | | | | |
| Harrell's C-index (95% CI) | 0.757 (0.753, 0.760) | 0.766 (0.755, 0.775) | 0.773 (0.744, 0.802) | 0.802 (0.774, 0.823) | 0.752 (0.734, 0.771) | 0.755 (0.727, 0.793) | 0.727 (0.684, 0.767) | 0.757 (0.707, 0.791) | 0.753 (0.725, 0.785) | 0.721 (0.679, 0.764) |
| Mean Calibration (95% CI) | 0.80 (0.78, 0.81) | 1.06 (1.02, 1.1) | 0.84 (0.75, 0.96) | 1.18 (1.07, 1.33) | 1.00 (0.91, 1.05) | 1.11 (0.99, 1.28) | 1.18 (0.97, 1.43) | 0.91 (0.79, 1.06) | 0.89 (0.74, 1.09) | 1.06 (0.91, 1.22) |
| **PREVENT Base Equation – HF Outcome** | | | | | | | | | | |
| Harrell's C-index (95% CI) | 0.805 (0.801, 0.808) | 0.824 (0.815, 0.833) | 0.801 (0.77, 0.834) | 0.846 (0.821, 0.865) | 0.816 (0.799, 0.828) | 0.804 (0.772, 0.837) | 0.813 (0.773, 0.855) | 0.827 (0.761, 0.869) | 0.820 (0.797, 0.85) | 0.747 (0.689, 0.801) |
| Mean Calibration (95% CI) | 0.87 (0.86, 0.89) | 1.33 (1.27, 1.39) | 0.96 (0.82, 1.10) | 1.54 (1.39, 1.74) | 1.12 (1.02, 1.20) | 1.37 (1.19, 1.61) | 1.94 (1.56, 2.53) | 1.15 (1.01, 1.33) | 1.25 (1.04, 1.61) | 1.72 (1.43, 2.19) |
| **PREVENT Full Equation – HF Outcome** | | | | | | | | | | |
| Harrell's C-index (95% CI) | 0.808 (0.805, 0.811) | 0.831 (0.822, 0.839) | 0.817 (0.789, 0.846) | 0.855 (0.830, 0.879) | 0.824 (0.808, 0.837) | 0.803 (0.769, 0.839) | 0.814 (0.777, 0.848) | 0.834 (0.757, 0.867) | 0.821 (0.800, 0.858) | 0.754 (0.698, 0.808) |
| Mean Calibration (95% CI) | 0.66 (0.65, 0.67) | 1.14 (1.09, 1.18) | 0.90 (0.78, 1.04) | 1.22 (1.10, 1.38) | 1.03 (0.95, 1.10) | 1.14 (1.00, 1.34) | 1.55 (1.24, 2.02) | 0.94 (0.82, 1.08) | 1.04 (0.88, 1.38) | 1.42 (1.17, 1.78) |
| Abbreviations: NH = non-Hispanic; NHPI = Native Hawaiian or Pacific Islander; SE = South East; CI = confidence interval | | | | | | | | | | |

© 2026 Au M et al. *JAMA Network Open.*
