# Supplementary material for: PREVENT Equation Performance in Asian and Native Hawaiian and Other Pacific Islander Groups
Source: JAMA Netw Open. 2026 Feb 12;9(2):e2556915. doi: 10.1001/jamanetworkopen.2025.56915 (PMC12902880; doi:10.1001/jamanetworkopen.2025.56915)
Supplement: Supplement 2. — Data Sharing Statement [file jamanetwopen-e2556915-s002.pdf]

Data Sharing Statement

Au. PREVENT Equation Performance in Asian and Native Hawaiian and Other Pacific Islander Groups. *JAMA Netw Open*. Published February 12, 2026.
doi:10.1001/jamanetworkopen.2025.56915

Data
Data available: No

Additional Information
Explanation for why data not available: Individual-level patient data will not be shared.
